# Treatment of Rats with a Self-Selected Hyperlipidic Diet, Increases the Lipid Content of the Main Adipose Tissue Sites in a Proportion Similar to That of the Lipids in the Rest of Organs and Tissues

**DOI:** 10.1371/journal.pone.0090995

**Published:** 2014-03-06

**Authors:** María del Mar Romero, Stéphanie Roy, Karl Pouillot, Marisol Feito, Montserrat Esteve, María del Mar Grasa, José-Antonio Fernández-López, Marià Alemany, Xavier Remesar

**Affiliations:** 1 Department of Nutrition and Food Science, Faculty of Biology, University of Barcelona, Barcelona, Spain; 2 Institute of Biomedicine, University of Barcelona, Barcelona, Spain; 3 CIBER (Centro de Investigación Biomédica en Red) OBN (Obesidad y Nutrición), Institute of Health Carlos III, Madrid, Spain; Northeast Ohio Medical University, United States of America

## Abstract

Adipose tissue (AT) is distributed as large differentiated masses, and smaller depots covering vessels, and organs, as well as interspersed within them. The differences between types and size of cells makes AT one of the most disperse and complex organs. Lipid storage is partly shared by other tissues such as muscle and liver. We intended to obtain an approximate estimation of the size of lipid reserves stored outside the main fat depots. Both male and female rats were made overweight by 4-weeks feeding of a cafeteria diet. Total lipid content was analyzed in brain, liver, *gastrocnemius* muscle, four white AT sites: subcutaneous, perigonadal, retroperitoneal and mesenteric, two brown AT sites (interscapular and perirenal) and in a pool of the rest of organs and tissues (after discarding gut contents). Organ lipid content was estimated and tabulated for each individual rat. Food intake was measured daily. There was a surprisingly high proportion of lipid not accounted for by the main macroscopic AT sites, even when brain, liver and BAT main sites were discounted. Muscle contained about 8% of body lipids, liver 1–1.4%, four white AT sites lipid 28–63% of body lipid, and the rest of the body (including muscle) 38–44%. There was a good correlation between AT lipid and body lipid, but lipid in “other organs” was highly correlated too with body lipid. Brain lipid was not. Irrespective of dietary intake, accumulation of body fat was uniform both for the main lipid storage and handling organs: large masses of AT (but also liver, muscle), as well as in the ”rest” of tissues. These storage sites, in specialized (adipose) or not-specialized (liver, muscle) tissues reacted in parallel against a hyperlipidic diet challenge. We postulate that body lipid stores are handled and regulated coordinately, with a more centralized and overall mechanisms than usually assumed.

## Introduction

Obesity and overweight are characterized by the excessive accumulation of fat as body reserve stored in a number of locations of white adipose tissue (WAT). Most of the fat is contained in large vacuoles in specialised cells, the adipocytes, that constitute the bulk of WAT [1], but many other types of cells contain lipid granules [2], which help fuel their own metabolism, whereas it is assumed that the main purpose of adipocyte = s fat is to supply energy to the whole organism under conditions of scarcity of energy [3].

The growing awareness of the importance of WAT as key metabolic controller in addition to its energy storage function [4], [5] has prompted the publication of an increasing number of papers on the mechanisms through which WAT hypertrophic alteration can explain, at least in part, the metabolic derangements of obesity [6], [7]. These studies, often do not take into account the differences in size, irrigation, cell types and metabolic activity between different WAT sites [8].

Total lipid content of a typical i.e.(Wistar) rat is in the range of 10–15% of its body weight [9], [10]. Energy storage lipids (i.e. triacylglycerols, TAG) account for more than 90% of these lipids, and in proportions even higher in the obese. It is generally accepted that the largest share of body lipid (TAG) is stored in well-defined WAT masses [11]. However, in previous studies using normal weight rats, we found that the careful dissection of all macroscopically distinguishable WAT masses accounted for about half [12] of body lipid [9]. This discordance means that lipid (mainly TAG) storage is distributed in a sizeable proportion in other tissues/structures different from the main WAT sites. These sources are largely known, but have not been previously quantified. There is a significant presence of lipid in (and around) muscle fibres [13], [14], which is directly involved in supplying energy during exercise [15]. Liver also contains a sizeable proportion of lipid, directly related to the production and export of energy through lipoproteins [16]. Brown adipose tissue contribution is also significant in proportion to its weight [17], but its overall size is small [18]. However, the size and lipid concentration of these tissues combined may justify only a part of the gap between WAT lipid and whole body lipid content.

The metabolic and regulatory importance of smaller WAT depots (epicardic, perimuscular, perivascular, mammary, etc.) is been increasingly acknowledged [19], but their individual mass is always relatively small, almost negligible with respect to the whole body or even when compared to the anatomically well known main WAT masses. However, the sum of many small contributions may in itself result in a sizeable mass that may fulfil the obvious gap between WAT masses and body lipid.

The objective of this study was to determine the proportion and dynamics of this important lipid depot with respect to WAT and body mass. We used two variables to highlight whether this mass of lipid is susceptible to change: sex and exposure to an obesogenic diet.

## Results

### Effects on Body Weight

Table 1 shows the *in vivo* body weight change of the experimental groups during the 28-day period of study. There were significant (ANOVA) effects of sex and cafeteria feeding. In 28 days, females’ weight increased 32% (controls) or 53% (cafeteria), and the initially larger males increased 46% (controls) or 55% (cafeteria).

**Table 1:** Weights and intestinal content of lean and overweight Wistar rats.

	units	female control	male control	female cafeteria	male cafeteria	P values	
						sex	diet
rat weight in vivo (initial)	g	167 ±3	238 ±1	168 ±2	238 ±5	<0.0001	NS
rat weight in vivo (final)	g	220 ±4	348 ±5	257 ±9	369 ±11	<0.0001	0.0014
body weight increase	g	54 ±3	110 ±6	89 ±8	131 ±7	<0.0001	0.0002
gastrointestinal content	g	17.3 ±0.6	23.0 ±0.8	20.6 ±1.2	21.1 ±1.1	0.0041	NS
rat net weight	g	203 ±4	325 ±4	236 ±8	347 ±11	<0.0001	0.0013
sum of all organs+carcass	g	196 ±4	311 ±4	226 ±8	334 ±11	<0.0001	0.0018
weight unaccounted for^A^	g	8.1 ±0.7	13.9 ±1.1	8.9 ±2.8	13.1 ±1.6	0.0094	NS
	% of BW	4.13 ±0.2	4.38 ±0.31	3.82 ±0.41	3.87 ±0.19	NS	NS

Values are the mean ± sem of six different animals. Statistical significance of the differences between groups (two-way ANOVA); the only significant interaction between sex and diet was observed in the gastrointestinal content; NS = not statistically significant (P>0.05). BW = body weight (final rat net weight).

A Part of the blood and other fluids.

The intestinal contents, constituted a considerable proportion of *in vivo* body weight: 7.8% and 6.6%, respectively for normal-weight female and male; the data for cafeteria-fed rats were 8.0% and 5.7%. The effects of sex were statistically significant (but not those of diet, in spite of a significant interaction between both factors in this parameter).

The net weight of rat, as well as the sum of all its organs, was both significantly influenced by sex and diet. In the same way that final weight and body weight increase did. The >unaccounted for = weight corresponds to the blood and fluids shed (or water evaporated) not recovered during dissection, with values in the range of 4% of the *in vivo* weight (no significant differences were observed in the percentages).

### Body and Organ Lipid Content

Table 2 presents the weights and lipid content of the dissected organs, as well as the estimated total muscle mass of the rat for the four experimental groups. As expected neither sex nor did diet affected the weight and lipid content of the brain. Liver weight was affected by sex but not by diet; in all four groups liver was 3.5–3.8% of *in vivo* body weight, a figure remarkably constant that resulted in significant changes related to body size (i.e. sex). The lipid content of liver was higher in cafeteria than in control diet-fed rats, an effect that also showed an interaction with sex. Gastrocnemius muscle changes followed closely those of liver, i.e. muscle lipid content (at least in that muscle) was affected by both sex and diet.

**Table 2:** Weights and lipid content of selected organs and tissues of lean and overweight Wistar rats.

organ/tissue		units	female control	male control	female cafeteria	male cafeteria	P values	
							sex	diet
brain	weight	g	1.74 ±0.1	1.89 ±0.06	1.84 ±0.04	1.85 ±0.03	NS	NS
	lipid	mg/g	77 ±2	62 ±11	87 ±9	69 ±8	NS	NS
		g	0.13 ±0.01	0.11 ±0.02	0.16 ±0.02	0.13 ±0.01	NS	NS
liver	weight	g	8.25 ±0.22	13.2 ±0.54	9.11 ±0.44	13.7 ±0.49	<0.0001	NS
	lipid	mg/g	43 ±1	41 ±1	50 ±3	59 ±3	NS	<0.0001
		g	0.35 ±0.01	0.54 ±0.03	0.46 ±0.03	0.82 ±0.07	<0.0001	<0.0001
gastrocnemius muscle	weight	g	1.10 ±0.06	2.10 ±0.05	1.24 ±0.07	2.02 ±0.06	<0.0001	NS
	lipid	mg/g	29 ±2	32 ±3	48 ±5	36 ±4	NS	0.0053
		mg	32 ±3	67 ±8	60 ±7	72 ±8	0.0026	0.0252
*calculated muscle mass*	*weight*	*g*	*79.2* ±*1.2*	*127* ±*2.2*	*81.7* ±*1.1*	*127* ±*2.9*	*<0.0001*	*NS*
	*lipid*	*g*	*2.30* ±*0.13*	*4.00* ±*0.39*	*3.92* ±*0.35*	*4.55* ±*0.51*	*0.0052*	*0.0084*
interscapular BAT	weight	g	0.50 ±0.04	0.53 ±0.06	0.69 ±0.07	0.77 ±0.09	NS	0.0046
	lipid	mg/g	305 ±40	447 ±27	502 ±29	523 ±17	0.0118	0.0002
		g	0.21 ±0.03	0.24 ±0.03	0.35 ±0.04	0.40 ±0.04	NS	0.0004
perirenal BAT	weight	g	0.52 ±0.08	0.71 ±0.12	0.59 ±0.08	0.87 ±0.07	0.0163	NS
	lipid	mg/g	416 ±70	522 ±36	538 ±20	580 ±28	NS	0.0491
		g	0.23 ±0.07	0.37 ±0.06	0.32 ±0.05	0.49 ±0.03	0.0101	NS
subcutaneous WAT	weight	g	6.65 ±0.71	11.4 ±0.80	11.5 ±2.55	17.5 ±2.12	0.0057	0.0055
	lipid	mg/g	577 ±50	576 ±58	677 ±65	652 ±34	NS	NS
		g	3.95 ±0.64	6.55 ±0.79	8.38 ±2.50	11.4 ±1.45	NS	0.0067
retroperitoneal WAT	weight	g	2.95 ±0.23	6.97 ±0.77	6.67 ±0.98	10.9 ±0.63	<0.0001	<0.0001
	lipid	mg/g	831 ±18	851 ±22	844 ±26	877 ±10	NS	NS
		g	2.44 ±0.15	5.93 ±0.70	5.64 ±0.91	9.52 ±0.47	<0.0001	<0.0001
mesenteric WAT	weight	g	3.76 ±0.42	4.73 ±0.37	6.45 ±0.77	6.80 ±0.90	NS	0.0017
	lipid	mg/g	738 ±37	777 ±17	771 ±35	794 ±34	NS	NS
		g	2.85 ±0.44	3.70 ±0.31	4.93 ±0.54	5.41 ±0.76	NS	0.0022
perigonadal WAT	weight	g	6.48 ±0.89	5.98 ±0.44	14.1 ±2.06	9.10 ±0.57	0.0299	0.0002
	lipid	mg/g	828 ±8	839 ±7	838 ±9	846 ±5	NS	NS
		g	5.38 ±0.77	5.01 ±0.36	11.87 ±1.83	7.70 ±0.50	0.0410	0–0003

Values are the mean ± sem of six different animals. Statistical significance of the differences between groups (two-way ANOVA); NS = not statistically significant (P>0.05). Data for calculated muscle mass are an extrapolation to total body muscle, calculated from net lean body mass and the data for gastrocnemius muscle. These data were not used for further calculations.

Table 2 also includes an extrapolation of possible total muscle lipid content based on the data for gastrocnemius muscle and the estimation of muscle mass from net lean body weight. These data were not used independently, since all muscle was included in the “other organs” fraction; however, the data presented show that the probable muscle lipid contribution to whole body lipid may be considerable, i.e., much higher than that of liver. The estimated data showed that muscle mass was affected by sex and its total lipid by diet.

Perirenal BAT weight was affected by sex but not by diet, which reflected in its overall lipid content; interscapular BAT, however, was deeply influenced by diet, but sex also resulted in a considerable increase in lipid content as a proportion of tissue weight. The proportion of lipid per unit of WAT weight was not significantly affected in any of the WAT sites by sex or diet. Thus, increase in lipid content was largely due to increased tissue mass. Subcutaneous WAT contained the lowest proportion of lipid versus weight (57% in both female and male rats) in the four sites studied, followed by mesenteric and both retroperitoneal and perigonadal masses, which in cafeteria-fed males showed mean values of up to 88% of lipid versus tissue weight.

Table 3 shows the total body lipid content of the rats and its distribution. Body lipid was affected by both sex and diet, male and cafeteria groups values being higher the female and control diet. The combined four-site WAT figure shows significant changes induced by diet, both in tissue weight relative to body weight and absolute lipid content, with no statistically significant effects due to sex in lipid content, and no significant changes in the tissue percentage of body lipids induced by either factor. The pattern for BAT was different; total lipid was affected by sex, but not by diet, and its share of body weight and lipids was unaffected by diet or sex.

**Table 3:** Body lipid distribution in lean and overweight Wistar rats.

	units	female control	male control	female cafeteria	male cafeteria	P values	
						sex	diet
total body lipid (BL)	g lipid	27.6 ±2.6	40.2 ±3.5	52.0 ±6.6	63.2 ±6.4	0.0298	0.0002
	% BW	14.84±1.20	13.3±1.23	23.6±2.72	19.9±1.47	NS	0.0003
WAT main four sites	g lipid	19.8 ±2.0	29.1 ±2.2	38.7 ±5.4	44.2 ±3.9	NS	0.0002
	% BW	10.0±0.86	9.17±0.67	16.3±2.04	11.6±0.27	0.0264	0.0013
	% BL	52.4 ±2.6	52.8 ±0.9	58.3 ±2.5	54.7 ±3.0	NS	NS
BAT main two sites	g lipid	0.52 ±0.08	0.71 ±0.12	0.59 ±0.08	0.87 ±0.07	0.0163	NS
	% BW	0.51±0.044	0.38±0.043	0.53±0.029	0.54±0.037	0.017	NS
	% BL	1.64 ±0.22	1.51 ±0.15	1.30 ±0.08	1.47 ±0.16	NS	NS
estimated muscle mass	% BL	8.82 ±1.13	9.98 ±0.55	8.14 ±1.07	7.28 ±0.69	NS	NS
liver	% BL	1.31 ±0.09	1.44 ±0.20	0.94 ±0.12	1.35 ±0.15	NS	NS
brain	% BL	0.51 ±0.06	0.31 ±0.07	0.34 ±0.07	0.21 ±0.03	0.0121	0.0353
rest of body lipid A	g lipid	12.1±1.65	17.7±2.12	19.9±2.01	27.3±3.15	0.015	0.0012
	% BW	5.98±0.55	5.51±0.45	8.41±0.76	7.93±0.85	NS	0.0017
	% BL	43.8±5.59	44.1±4.15	38.3±4.61	43.3±4.76	NS	NS

Values are the mean ± sem of six different animals. Statistical significance of the differences between groups (two-way ANOVA); NS = not statistically significant (P>0.05).

A Body lipid (BL) content of the rat minus the lipid content of the 4 WAT, 2 BAT sites, liver and brain.

An approximate estimation of muscle mass, based on the gastrocnemius data, showed no significant changes, but muscle accounted for a substantial 7–10% of total body lipid.

Liver lipid proportion versus total body lipids (1–1.5%) was not affected by sex or diet. However, brain lipids percentage of total body lipids changed with both sex and diet, with a share of 0.2 to 0.5% of total lipid content of the rat.

**Figure 1:**
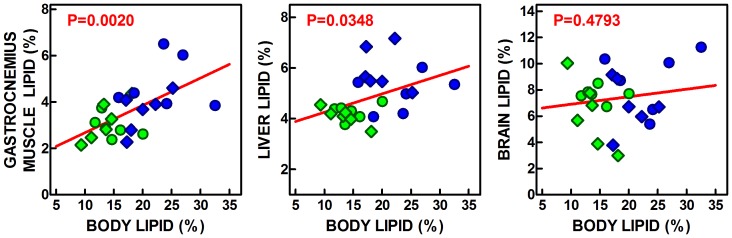
Correlation plots of gastrocnemius muscle, liver and brain lipid content (as % of tissue weight) versus the percentage of body lipid in Wistar rats fed a control or simplified cafeteria diet for 28 days. Each symbol represents an individual rat. Circles = females; Diamonds = males; Green = control diet, Blue = cafeteria diet. The red line shows the overall linear correlation, and the red figure indicates the statistical significance of the correlation.

**Figure 2:**
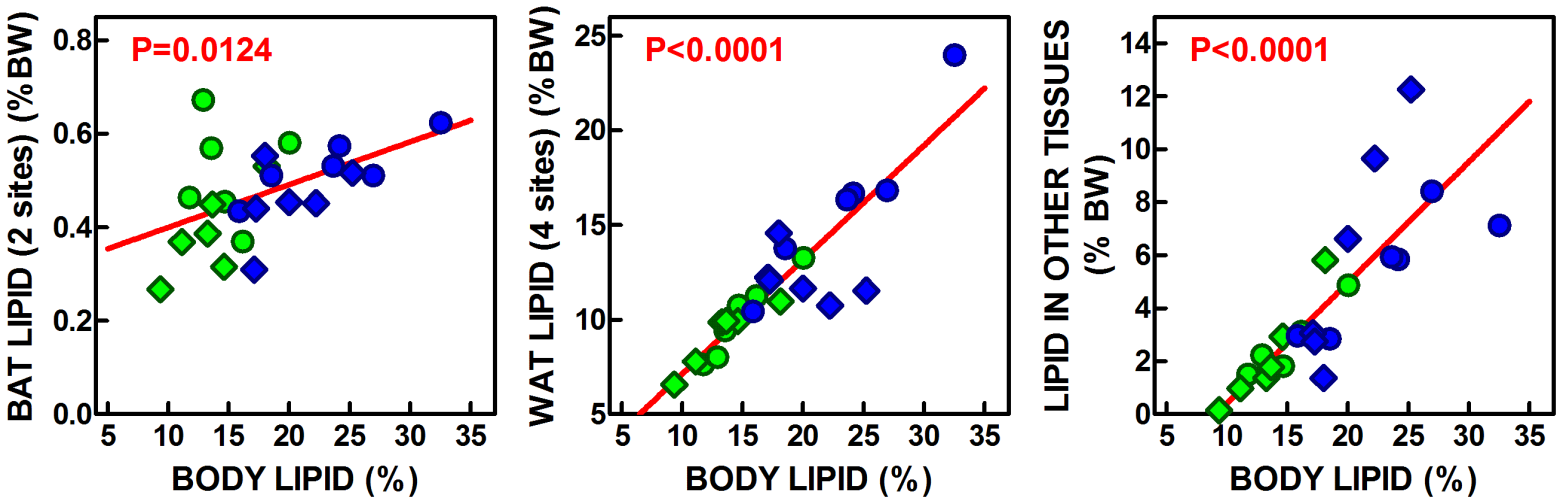
Correlation plots of lipid content (as % of body lipids of the combines values for two BAT sites, 4 WAT sited and “other tissues”) versus the percentage of body lipid in Wistar rats fed a control or simplified cafeteria diet for 28 days. The “other tissues” combined data represent the sum of body lipids minus those in brain, liver and the 2 sites of BAT and 4 sites of WAT studied. These data include other WAT, and BAT sites, as well as muscle, skin and all other organs. BW = body weight. Each symbol represents an individual rat. Circles = females; Diamonds = males; Green = control diet, Blue = cafeteria diet. The red line shows the overall linear correlation, and the red value indicates the statistical significance of the correlation.

The calculations done for (each rat) lipid content showed that 38–44% of total body lipid was not accounted for by the four WAT, two BAT sites, liver and brain. This value was not changed by diet or sex: but it was affected by both factors when analyzed in absolute terms. These values indicate that, in parallel with liver, BAT and WAT, the lipid content of the rest of the body was also directly related to total body lipid, as were the contents of liver and gastrocnemius muscle, but not of brain (Figure 1). When the lipid content of BAT, WAT and all other organs (not including the six adipose tissue sites analyzed separately, liver and brain, but including muscle, other adipose tissue sites and disperse cells, as well as skin and the remaining organs and tissues), we obtain the graphs presented in Figure 2. In spite of its small size and the fact that only two locations were studied, BAT proportion of total body lipids was significantly correlated with this latter figure. The correlation was more marked for the combined four WAT sites analyzed, which lipid content was highly correlated with total body lipids. The same can be said of the other organs’ lipid, which contribution and direct lineal correlation with total body lipid showed a marked parallelism with the lipid present in adipose tissue main masses.

### Dietary Lipid Handling

Table 4 presents the differences in dietary lipid handling by rats and its relations to sex and diet. The total net (i.e. without intestinal contents) energy content (bomb calorimeter) of the rats was significantly affected by sex and diet. Total rat lipid energy content was also affected in the same way, however, the expression of body lipids as percent of total body energy was dependent on diet but not on sex; the non-lipid (essentially protein) body energy content, however, was related significantly to sex but not to diet.

**Table 4:** Dietary lipid energy handling by Wistar rats, effect of diet and sex.

	units	female control	male control	female cafeteria	male cafeteria	P values	
						sex	diet
rat body energy	MJ	4.85±0.22	7.79±0.41	6.85±0.38	9.26±0.37	<0.0001	<0.0001
rat lipid energy content	MJ	1.02±0.10	1.49±0.13	1.92±0.24	2.34±0.24	0.0298	0.0002
	% energy	21.1±1.96	19.1±2.11	28.1±2.66	25.3±2.44	NS	0.0097
rat body non lipid energy	MJ	3.94±0.41	6.15±0.55	4.76±0.46	6.83±0.71	0.0008	NS
dietary energy intake	MJ/28 d	6.56±0.48	8.87±0.69	14.5±1.33	19.6±1.59	0.0035	<0.0001
	W	2.00±0.09	3.22±0.17	2.83±0.16	3.83±0.15	<0.0001	<0.0001
	W/kg BW	9.10±0.32	9.24±0.40	10.9±0.26	10.4±0.14	NS	<0.0001
lipid dietary energy intake	MJ	0.64±0.08	0.85±0.09	5.19±0.65	6.27±0.73	NS	<0.0001
	% energy intake	9.68±0.68	9.68±0.75	35.7±2.98	32.1±3.33	NS	<0.0001

Values are the mean ± sem of six different animals. Statistical significance of the differences between groups (two-way ANOVA); NS = not statistically significant (P>0.05).

Dietary energy intake was, again, affected by sex and diet, but the net flow of energy per unit of body weight was not affected by sex, suggesting that the smaller size of female rats justified the significance of the differences related to sex but not to diet. Lipid energy intake was not affected by sex and deeply by diet, as expected.

**Figure 3:**
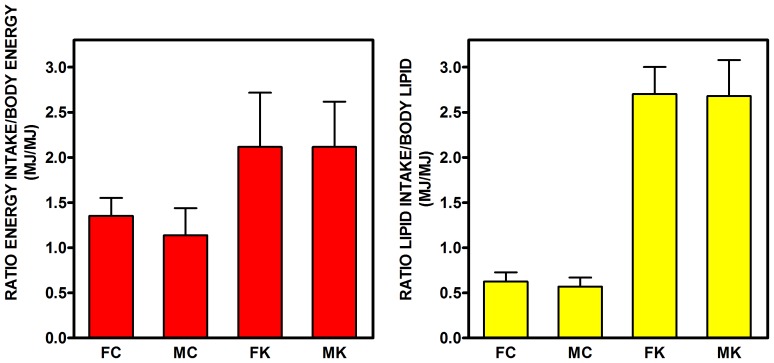
Ratios of energy and lipid intake with respect to whole body energy and lipid content in rats fed a control or simplified cafeteria diet for 28 days. FC = female control; MC = male control; FK = female cafeteria; MK = male cafeteria. Each point corresponds to the mean ± sem of six different animals (data for food/lipid intake, N = 3 (2-rat cages), data for body energy and lipid N = 6). Statistical significance of the differences (2-way-ANOVA): Energy: P<0.001 for diet, NS (i.e. P>0.05) for sex; Lipid: P<0.0001 for diet, NS for sex.

The ratios of ingested energy/body energy, and ingested lipid energy/body lipid energy for the 28-day period studied are shown in Figure 3. Cafeteria feeding almost doubled the ratio of energy ingested in 28 days versus body energy, with no significant influence of sex. The ratio of lipid intake versus body lipid was more than four-fold higher in cafeteria-fed rats, again with no effects of sex.

## Discussion

Changes in body weight under exposure to a self-selected hypercaloric (hyperlipidic) cafeteria diet were in agreement with previous results [20], with effects, in part, more marked on females [21]. An unexpected result from our study was the finding that the proportion of *in vivo* body weight that corresponds to intestinal contents was in the range of 6–8% of body weight, but this percentage was not affected by diet in spite of the higher amount of food taken up by cafeteria-fed rats. This factor should be taken into account when analyzing effects of treatments on body weight.

Most of body lipids are triacylglycerols, which main function is the storage of energy [22], but lipids as a whole also include neutral sphingolipids, cholesterol and phospholipids, as well as smaller amounts of glycolipids and other complex or rare lipids. The body content of most of these components is directly linked to their presence in membranes, with nervous tissue (myelin) as its maximal exponent. The total body content of cholesterol in a Wistar rat is in the range of 2 g/kg [23], i.e. about 1.5% of body lipids. Phospholipid content ranges from 40 mg/g in brain [24] to 6 mg/g in muscle [25] or 4 mg/g in WAT [26]. A rough estimation of both cholesterol and phospholipid data combined results in about 10–12 mg/g, representing less than 8% of body lipids in a normal-weight adult rat, and obviously much less in obese animals, with their heavy load of triacylglycerols. A large portion of structural lipids are concentrated in brain, which lipid content was not affected by dietary treatment, and which showed no correlation with total body lipids.

The results presented here show that there is a surprisingly high proportion of lipid (more than 40% of total body lipids) not accounted for by the main macroscopic WAT sites, even when brain, liver and BAT main sites were discounted. This sole datum suggests that our usual perception of well defined large WAT masses as main fat (energy) storage site could no longer be maintained.

It can be questioned; however, whether the large mass of lipid found outside the four main WAT sites has a role for energy storage, or even if it corresponds actually to adipose tissue. All cells contain lipids, but only a few types contain a significant amount of triacylglycerols as energy reserve or storage depot. There are no significant masses of phospholipid, other complex lipids or cholesterol (i.e. all non-triacylglycerol or reserve lipids) in any organ, with the exception of nervous tissue which contains large proportions of these (mainly glycosphingolipids) in myelin [27]; for this reason, brain was analyzed separately. All other phospholipids and cholesterol in the body are essentially components of membranous structures of the cells [28]. Their global estimation is difficult, because of small differences in organelle, type of cell and modifications induced by diet and physiological state [29]. Adipose tissue content of phospholipid is fairly small [30] with respect to its large lipid content, practically made up only by triacylglycerols. Since adipose tissue can be found interspersed within other tissues and organs, and the large amounts of “unaccounted for” lipids could not be attributed to structural lipids we can safely assume that, most of the lipid in “other organs or tissues” is constituted mainly by triacylglycerols.

A very rough estimate of muscle lipid contribution (in the range of 8% of body lipids) includes both intramyofibrilar lipid [31] and interspersed adipose tissue cells [32], the latter being probably the major component. By extension, and taking into account the large number of small weight WAT and BAT secondary sites: pericardic, other mediastinal, perivascular, bone marrow, intestinal cordons, etc. we can safely assume that most of the lipid in “other organs or tissues” is probably made up of adipose cells (WAT, BAT or brite adipose cells).

The peculiar behaviour of body lipid reserves in front of the dietary challenge of a self-selected hyperlipidic diet results in the data presented elsewhere [33], [34] and in part repeated here on WAT masses increase, higher body lipid content and sex-related differences. However, in the present study we have analyzed the relative mass of the sum of all that “disperse” adipose tissue, and found that it is very closely correlated to total body lipid, in the same way that liver, 2-site BAT, but especially 4-site WAT lipids do. The high correlation between all these parameters clearly indicate that body energy storage is centrally regulated, and body lipids increase (in widely different compartments) in parallel to higher dietary energy (lipid) availability. In normally-fed animals (overweight rats are in the limit of normalcy: the rats used here were not yet obese), the pattern of fat deposition was maintained in all organs, except for the brain, which mass of lipid is not used for the storage of energy and thus is unrelated to dietary or total body lipid.

In spite of the enormous load of lipid ingested with respect to body energy, the large intake affected less body energy than expected, in a way that non-lipid (i.e., protein) constitutes the largest fraction of body energy in spite of cafeteria-fed rats ingesting twice the equivalent of their body energy in 28 days (and four-fold their lipid stores) than chow-fed controls. This is compensated by an efficient increase of energy output [35], since the actual storage of this extra energy is, necessarily, only a fraction of the total energy (and especially of lipid energy) ingested [36].

In sum, we have found that, largely irrespective of dietary lipid/energy intake, the accumulation of body fat was fairly uniform both for the main lipid storage and handling organs: large masses of WAT, liver, muscle, BAT, as well as by the smaller masses of BAT, WAT and interspersed adipose cells in various tissues. These storage sites, large and small, in storage-specialized (adipose) or not-specialized (liver, muscle) tissues reacted in the same way, increasing their size in parallel in front of a hyperlipidic diet challenge. In consequence, the assumption that large WAT masses contain most of the body fat reserves and their changes are controlled in a different way than in “disperse” adipose tissue or intracellular lipid droplets in liver and muscle could no longer be sustained. We postulate that body lipid stores are handled and regulated in a coordinated way, with a more centralized and general regulatory mechanisms than usually assumed.

## Materials and Methods

### Ethics Statement

All animal handling procedures and the experimental setup were carried out in accordance with the animal handling guidelines of the European, Spanish and Catalan Authorities. The Committee on Animal Experimentation of the University of Barcelona authorized the specific procedures used.

### Animals and Diets

Sixty-day old female and male Wistar rats (Harlan Laboratories Models, Sant Feliu de Codines, Spain) were used. The rats were maintained under standard conditions (21–22°C, 50–60% relative humidity, and 12 h light/dark cycle) in two-rat cages lo limit isolation stress. Half of the rats (6 male and 6 female) were maintained under these conditions, and fed *ad libitum* with standard rat chow (maintenance type A04, Panlab, Barcelona, Spain) and tap water for 28 days. The other half, 6 female and 6 male, were given a simplified cafeteria diet (plain cookies, liver pâté, bacon and milk with 25% sugar) *ad libitum* [37] in addition to the standard chow (and tap water) for 28 days; at the end of the period, the cafeteria-fed rats were already overweight as previously described [37].

### Dissection and Organ Extraction

At the end of the 28-day period, the animals were killed by decapitation and immediately dissected; blood was recovered in dry heparinised beakers. All tissue samples were blotted and weighed, then frozen in liquid nitrogen, and kept at −70°C for later use in analyses. After shedding part of the blood, the rats were eviscerated, and the weights of their stomach, small and large intestine contents, as well as those of liver, brain, heart, and left gastrocnemius muscle, were determined. Brown adipose tissue (BAT) masses in the interscapular and perirenal sites were dissected, cleaned of WAT, weighed and frozen. WAT masses of mesenteric/omental, retroperitoneal (plus perirenal), perigonadal (epididymal or periovaric and annexes) were also dissected, weighed and frozen. The rat was skinned and all macroscopic subcutaneous WAT was removed, including the gluteal masses, the inguinal fat pads, the axillary and interscapular WAT; this combined mass of WAT was considered subcutaneous WAT, which was weighed and then mixed before freezing and sampling. Skin (with hair) was also weighed.

Rat *net* weight was the *in vivo* weight (before killing) minus the contents of the guts [38] (Table 1). The detailed sum of all anatomical fractions of the rat did not add up to the net rat weight because of the inescapable loss of blood and fluids during the dissection process (taking about 90 minutes per animal).

Lean body mass was calculated as the net rat weight minus the four main macroscopic WAT sites; this lean body mass was used for an approximate estimation of total body muscle assuming that this proportion was constant, and comparable to that measured [39] in animals of similar characteristics; the corresponding factor applied was: 43% of net lean body weight.

All organs, except the samples taken of brain, heart, gastrocnemius muscle, liver, BAT and WAT (whose weights were known) were reunited, after weighing, with the skin and the remaining carcass (including the blood recovered) in a plastic bag, to be later weighed and frozen. Prior to analysis, the sealed bags were autoclaved and minced with an industrial-type blender to obtain a fine paste [10], which was used for lipid analysis and energy content using an IKA C7000 (IKA, Staufen, Germany) adiabatic bomb calorimeter. Food energy content was measured likewise.

### Lipid Analysis

Minced samples of tissues or carcass paste (about 0.5 g) were extracted for 24 h in rotary mixers with 10 mL of trichloromethane: methanol (2∶1 v/v) in Teflon-lined screw-cap tubes at room temperature. Total lipids were estimated in the organic phase, after repeated washings, by weight as described previously [40].

The lipid content for each of the tissues analyzed was calculated from their known weights and the proportion of lipid measured in the extracted samples. Total body lipid content was calculated for each individual rat by adding up the lipids in the paste and those found in the analyzed tissue samples Thus, the only part not included in the analysis was the blood and fluids lost in the dissection process (Table 1).

### Statistics

Statistical analyses were carried out using a two-way ANOVA program (Prism 4, GraphPad Software, La Jolla, CA USA), with the variables of sex and cafeteria feeding. Standard linear correlations were analyzed using the same program.
